# TagSeq for gene expression in non‐model plants: A pilot study at the Santa Rita Experimental Range NEON core site

**DOI:** 10.1002/aps3.11398

**Published:** 2020-11-22

**Authors:** Hannah E. Marx, Stephen Scheidt, Michael S. Barker, Katrina M. Dlugosch

**Affiliations:** ^1^ Department of Ecology and Evolutionary Biology University of Arizona Tucson Arizona 85721 USA; ^2^ Department of Ecology and Evolutionary Biology University of Michigan Ann Arbor Michigan 48109‐1048 USA; ^3^ Howard University 2400 6th Street NW Washington D.C. 20059 USA; ^4^ Solar System Exploration Division NASA Goddard Space Flight Center Greenbelt Maryland 20771 USA; ^5^ Center for Research and Exploration in Space Science and Technology NASA Goddard Space Flight Center Greenbelt Maryland 20771 USA

**Keywords:** gene expression, National Ecological Observatory Network (NEON), RNA‐seq, Sonoran Desert, TagSeq, transcriptome

## Abstract

**Premise:**

TagSeq is a cost‐effective approach for gene expression studies requiring a large number of samples. To date, TagSeq studies in plants have been limited to those with a high‐quality reference genome. We tested the suitability of reference transcriptomes for TagSeq in non‐model plants, as part of a study of natural gene expression variation at the Santa Rita Experimental Range National Ecological Observatory Network (NEON) core site.

**Methods:**

Tissue for TagSeq was sampled from multiple individuals of four species (*Bouteloua aristidoides* and *Eragrostis lehmanniana* [Poaceae], *Tidestromia lanuginosa* [Amaranthaceae], and *Parkinsonia florida* [Fabaceae]) at two locations on three dates (56 samples total). One sample per species was used to create a reference transcriptome via standard RNA‐seq. TagSeq performance was assessed by recovery of reference loci, specificity of tag alignments, and variation among samples.

**Results:**

A high fraction of tags aligned to each reference and mapped uniquely. Expression patterns were quantifiable for tens of thousands of loci, which revealed consistent spatial differentiation in expression for all species.

**Discussion:**

TagSeq using de novo reference transcriptomes was an effective approach to quantifying gene expression in this study. Tags were highly locus specific and generated biologically informative profiles for four non‐model plant species.

Gene expression studies that involve sampling many individuals or tissues can be powerful for identifying variation in transcriptional activity and function (e.g., among populations, over time, in response to the environment or other treatments; Gould et al., 2018; Mead et al., 2019), as well as the structure of transcriptional networks and the genetic basis of gene expression variation (Wisecaver et al., 2017; Li et al., 2020). Such studies are of high interest for non‐model species responding to natural environments, as well as for model species (Matz, 2018; Zaidem et al., 2019). Quantitative next‐generation sequencing of expressed genes, known as RNA‐seq, has made expression studies broadly accessible for non‐model species, but remains expensive per sample and difficult to scale up for questions that require high numbers of replicates (Lohman et al., 2016).

A cost‐effective approach is to target only a small region of each transcript for sequencing, identifying and quantifying its expression while avoiding sequencing across its full length. Several versions of this approach have involved reading a short tag of sequence upstream of the poly(A) tail of mRNA. These methods have their roots in one of the first approaches to RNA sequencing prior to the next‐generation sequencing era, expressed sequence tags or ESTs (Parkinson and Blaxter, 2009). Meyer et al. (2011) developed an updated version for next‐generation applications, which continues to be used and adapted (e.g., Dixon et al., 2018; Kremling et al., 2018; Mitchell et al., 2019; Pallares et al., 2020). Recently, Lohman et al. (2016) published further developments of what has become known as TagSeq (also TAGseq or Tag‐seq) and compared its performance with standard RNA‐seq of full transcripts. Notably, they find that TagSeq achieves higher accuracy than standard RNA‐seq, presumably because sequencing effort is distributed more evenly to all transcripts when only the tag sequence is targeted.

TagSeq tags are short sequences, however, and must be aligned to a reference to fully identify the loci that are being expressed (Meyer et al., 2011). For non‐model species and multi‐species studies, high‐quality reference genomes are not likely to be available. Instead, assembled reference transcriptomes can be generated using standard RNA‐seq (Matz, 2018). Reference transcriptomes will differ from genomes in that not all loci will be represented by transcripts present in a given sample, not all transcripts will be assembled to full length, and the assembly will vary in the degree to which splice variants, alleles, and paralogs will occur as unique sequences or be merged (Meyer et al., 2011; Yang and Smith, 2013; Carpenter et al., 2019; Patterson et al., 2019). These issues will reduce the number of TagSeq reads that can be uniquely mapped to the reference, relative to a full genome, and they may be particularly problematic in plants where gene and genome duplications are common (Barker et al., 2016; One Thousand Plant Transcriptomes Initiative, 2019; Li and Barker, 2020), although sequencing at the variable 3′ untranslated region (UTR) should maximize locus discrimination (Rise et al., 2004).

Meyer at al. (2011) originally demonstrated the TagSeq method in a non‐model species of coral, where tags were aligned to a reference transcriptome. Many subsequent studies have successfully used a similar approach in other non‐model animals (e.g., Kenkel and Matz, 2016; Dixon et al., 2018; Kriefall et al., 2018; Rocker et al., 2019). In plants, however, TagSeq studies to date appear to have been confined to model species for which a high‐quality reference genome is available (Meyer et al., 2014; Des Marais et al., 2015; Lovell et al., 2016; Kremling et al., 2018; Chu et al., 2019; Razzaque et al., 2019; Weng et al., 2019). How TagSeq will perform using a reference transcriptome in plants is not clear given the lack of such studies and a paucity of relevant performance information for TagSeq.

Here we report a pilot study using TagSeq to quantify gene expression for four plant species, as part of a study of gene expression variation at the Santa Rita Experimental Range and National Ecological Observatory Network (NEON) core site (Green Valley, Arizona, USA). We assembled a reference transcriptome for each species using standard RNA‐seq and analyzed gene expression using TagSeq across multiple individuals for each species, sampled at two locations and three time points. We evaluated the fraction of tags that map uniquely to loci in the reference transcriptome, and the specificity of mapping against references from the same sample, from another sample of the same species, and from other species. We further evaluated the performance of TagSeq in terms of the number of reference loci observed as a function of TagSeq sequencing effort, and the variation in TagSeq profiles across species, sites, and times. Our goal was to assess whether TagSeq is a locus‐specific and biologically informative approach for non‐model species lacking a high‐quality reference genome.

## METHODS

### Sampling

**Figure 1:**
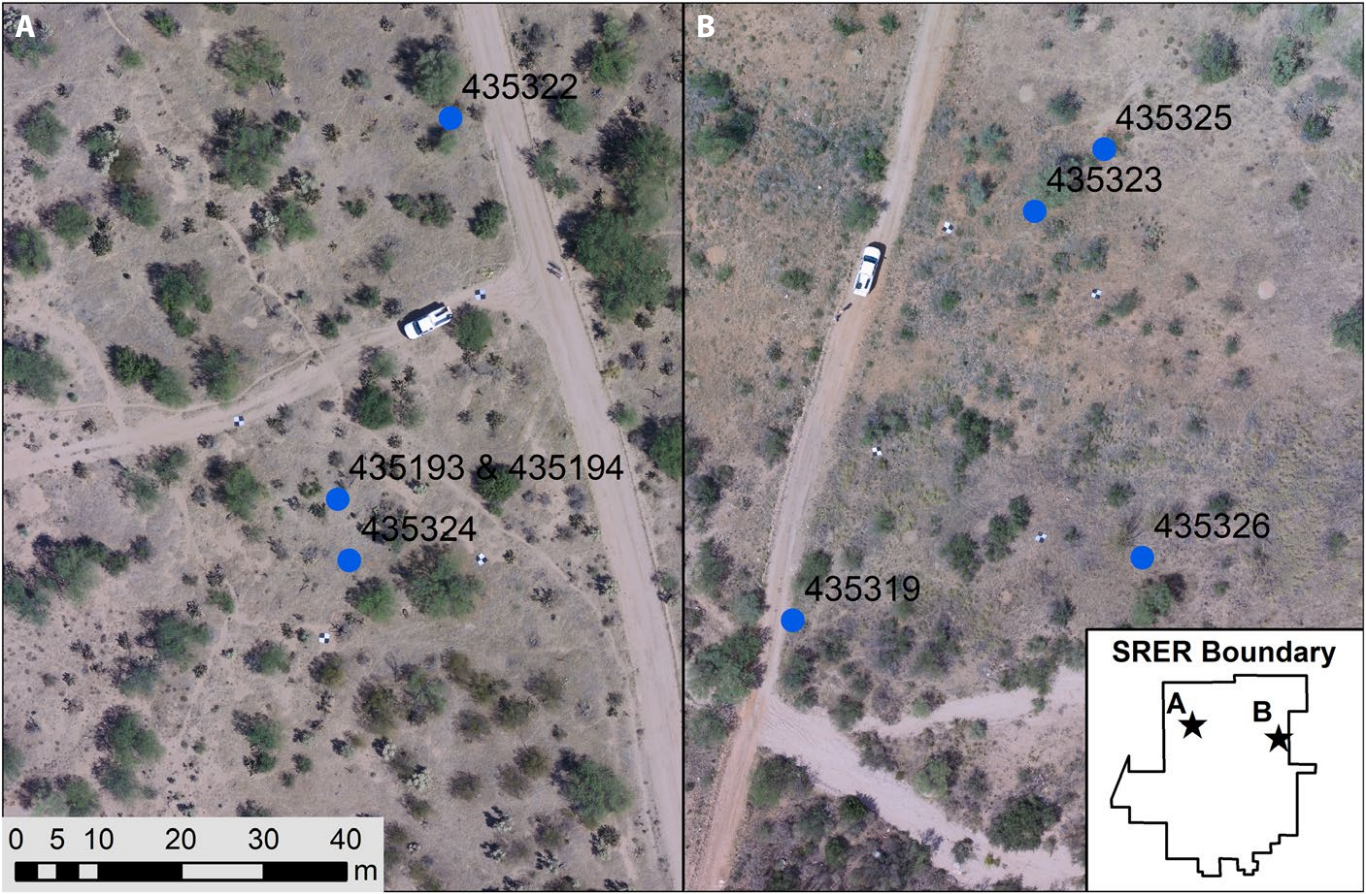
Aerial map of the Phone Pole (A) and Grassland (B) sampling areas. Blue circles indicate sampling locations (multiple samples per location), and labels indicate accession numbers for associated vouchers at the University of Arizona herbairum (ARIZ). Latitude, longitude, and sample details are given in Appendix 1.

Our pilot study focused on four commonly occurring species at the Santa Rita Experimental Range Long Term Research and NEON core site (SRER; Fig 1, Appendix 1). These include the native species *Tidestromia lanuginosa* (Nutt.) Standl. (Amaranthaceae; woolly tidestromia), *Parkinsonia florida* (Benth. ex A. Gray) S. Watson (Fabaceae; blue palo verde), and *Bouteloua aristidoides* (Kunth) Griseb. (Poaceae; needle grama), as well as the introduced species *Eragrostis lehmanniana* Nees (Poaceae; Lehmann lovegrass; native to southern Africa). All species were identified using a combination of the historical flora of the SRER (Medina, 2003), the Arizona Flora (Kearney et al., 1960), and the Flora of North America (Flora of North America Editorial Committee, 1993). Based on chromosome counts of these and congeners in the Chromosome Counts Database (Rice et al., 2015), we infer that *P. florida* and *T. lanuginosa* are likely diploid species, while the grasses *B. aristidoides* and *E. lehmanniana* are both likely to be tetraploids. Vouchers were deposited in the University of Arizona herbarium (ARIZ; Fig. 1, Appendix 1).

Tissue from mature plants was collected from an apparently healthy individual representing each target species weekly on three dates (September 1, 7, and 13) during the 2017 growing season. An entire stem was sampled for *B. aristidoides* (with flowers and fruits) and *E. lehmanniana* (without flowers or fruits). Only leaves and leaflets were sampled for *P. florida* and *T. lanuginosa*. At each sampling date, 2–4 individuals were sampled from each species at each of two locations (“Phone Pole” and “Grassland”; 4–6 samples total/species/date; Fig. 1, Appendix 1). For *P. florida*, samples at the same location and date were not from different individuals, but instead were multiple collections of tissue from the same individual (replicates). The same individual was also resampled at each time point for *P. florida*, and individuals from the same population were sampled for *B. aristidoides*, *E. lehmanniana*, and *T. lanuginosa*. Samples were collected in the same order on each day beginning at the Phone Pole location and as close as possible to the same time of day (afternoon). Leaf tissues were flash frozen in liquid nitrogen in the field and transported to the University of Arizona for RNA extraction. Total RNA was extracted from leaf tissue using the Spectrum Plant Total RNA Kit (Sigma‐Aldrich, St. Louis, Missouri, USA) following the manufacturer’s Protocol A.

The locations included a relatively undisturbed grassland dominated by native species (Grassland) and a more frequently disturbed location near research facilities (Phone Pole). The Grassland location spanned ~500 m along the south side of access Road 424 and was dominated by mostly native grasses (including *B. aristidoides*, *B. barbata* Lag. var. *rothrockii* (Vasey) Gould, and *B. repens* (Kunth) Scribn. & Merr.) and ocotillo (*Fouquieria splendens* Engelm.). The Phone Pole location followed a wash along the west side of Road 401 for ~300 m and was dominated by cacti (*Ferocactus wislizeni* (Engelm.) Britton & Rose, *Opuntia engelmannii* Salm‐Dyck, *Cylindropuntia fulgida* (Engelm.) F. M. Knuth), mesquite (*Prosopis velutina* Wooton), and creosote (*Larrea tridentata* (DC.) Coville). The Grassland was roughly 200 m higher in elevation than the Phone Pole location and typically receives greater annual moisture (M. McClaran, University of Arizona, personal communication).

### RNA‐seq for reference transcriptomes

A single sample for each species was selected to be used for both the RNA‐seq reference and a TagSeq expression sample (Appendix 1). RNA‐seq libraries were prepared and sequenced at the Arizona State University’s Biodesign Institute Genomics core facility. Total RNA was used to prepare cDNA using an Ovation RNA‐Seq System via single primer isothermal amplification (#7102‐A01; NuGEN, Redwood City, California, USA) and automated on an Apollo 324 liquid handler (Takara Bio, Kusatsu, Shiga, Japan). cDNA was quantified on a NanoDrop Lite (Thermo Fisher Scientific, Waltham, Massachusetts, USA) and was sheared to approximately 300‐bp fragments using an M220 ultrasonicator (Covaris, Woburn, Massachusetts, USA). Libraries were generated using the KAPA Biosystems Illumina library preparation kit (#KK8201; Roche, Basel, Switzerland). Fragments were end repaired and A‐tailed, and individual indexes and adapters (#520999; Bioo Scientific, Austin, Texas, USA) were ligated onto each sample. The adapter‐ligated molecules were cleaned using AMPure XP beads (#A63883; Beckman Coulter, Brea, California, USA) and amplified with the KAPA Biosystems HiFi enzyme (#KK2502; Roche). Each library was then analyzed for fragment size on an Agilent TapeStation 4200 (Agilent Technologies, Santa Clara, California, USA) and quantified using the KAPA ABI Prism qPCR Kit (#KK4835; Roche) on Thermo Fisher Scientific’s QuantStudio 5 before multiplex pooling (13–16 samples per lane in equal representation) and sequencing on the NextSeq 500 platform (paired‐end 2 × 150 bp High‐Output Kit; Illumina, San Diego, California, USA).

Raw reads were filtered and trimmed for adapters and low‐quality bases using SnoWhite version 2.0.3 (Dlugosch et al., 2013), including TagDust filtering (‐D; Lassmann et al., 2009), SeqClean filtering and trimming (‐L; Chen et al., 2007), and a minimum Phred score (‐Q) of 20. The cleaned read pairs were realigned using fastq‐pair (Edwards and Edwards, 2019). Transcripts were assembled using SOAPdenovo‐Trans (Xie et al., 2014) using an optimized *k*‐mer of 57 (Marx et al., 2020a) and archived at https://doi.org/10.5281/zenodo.3740232 (Marx et al., 2020b).

Several aspects of reference assembly quality were assessed. Summary statistics including the number of contig scaffolds, scaffold lengths, and N50 were calculated by TransRate version 1.0.3 (Smith‐Unna et al., 2016). The completeness of transcriptome coverage was quantified using BUSCO version 4.0.5 (Seppey et al., 2019), which identifies representation of a collection of universal single‐copy orthologs for the Viridiplantae (Viridiplantae Odb10) and the eukaryotes (Eukaryote Odb10). Finally, reference contigs matching known proteins were identified using TransPipe (Barker et al., 2010), in which contigs were compared to protein sequences from 25 sequenced and annotated plant genomes from Phytozome (Goodstein et al., 2012) using BLASTX (Wheeler et al., 2008). Best‐hit proteins were paired with each gene at a minimum cutoff of 30% sequence similarity over at least 150 sites. To determine the reading frame and generate estimated amino acid sequences, each gene was aligned against its best‐hit protein by GeneWise 2.2.2 (Birney et al., 2004). Based on the highest‐scoring GeneWise DNA–protein alignments, stop and ‘N’‐containing codons were removed to produce estimated amino acid sequences for each gene (archived at https://doi.org/10.5281/zenodo.3740232; Marx et al., 2020b).

### TagSeq gene expression

TagSeq libraries for all samples were prepared and sequenced at the University of Arizona Genomics core center. Total RNA was used to prepare TagSeq libraries according to the detailed protocol given in Lohman et al. (2016), with the DNase I step included (QIAGEN #79254; QIAGEN, Valencia, California, USA). RNA was fragmented using NEBNext RNA fragmentation buffer (New England Biolabs, Ipswich, Massachusetts, USA), cleaned using RNAClean XP beads (Beckman Coulter), and quantified using RNA PicoGreen (Life Technologies, Carlsbad, California, USA). cDNA was synthesized using forward primers with four degenerate bases near the 3' end (Eurofins Scientific, Luxembourg City, Luxembourg) for the identification of PCR duplicates, and then PCR amplified for 15 cycles, incorporating sample‐specific barcodes. PCR products were purified using AMPure XP beads (Beckman Coulter), and a Pippen Prep electrophoresis system (Sage Science, Beverly, Massachusetts, USA) was used for 400–500‐bp size selection. DNA was quantified using DNA PicoGreen (Life Technologies) and pooled in equal representation. The final library was quantified using the KAPA SYBR FAST ABI Prism qPCR Kit (Roche). A total of 56 samples (Appendix 1) were sequenced together on one lane of the NextSeq 500 platform (1 × 75 bp High‐Output Kit; Illumina). All primer sequences were unmodified from those given in Lohman et al. (2016).

Tag sequences were cleaned of several potential contaminants before analysis. PCR duplicates were identified as sequences that were identical over the first 57 bases, which included the four‐base degenerate primer region, three‐base GGG RNA priming region, and 50 additional bases of unique sequence (using the script ‘removePCRdups57'; Marx et al., 2020b). The program ‘cutadapt’ version 1.9.1 (Martin, 2011) was used to trim the 5′ degenerate primer region, 3′ poly(A) tails (eight or more bases), 3′ low‐quality bases (minimum score 20), and primer/adapter contaminants with a minimum overlap of 8 bp. Reads less than 57 bases after trimming were discarded. The remaining reads were considered unique sequence tags.

To quantify expression of each locus, tags were aligned to the reference transcriptomes using BWA‐mem version 0.7.17 (Li and Durbin, 2010) with a bandwidth of 5 bp (‐w 5; because gaps relative to the transcriptome reference are not expected in these tag sequences). All other parameters were set at the default value. The number of hits to a reference sequence (expressional level) was tallied using HtSeq‐count version 0.5.4 (Anders et al., 2015), with ‐‐stranded=no (the reference assembly is not stranded). A GTF file was generated from the transcriptome assembly for use with HtSeq‐count (using the script ‘create_GTF.pl’; Marx et al., 2020b). Hits to each locus were combined across samples and filtered for loci with a minimum of five hits across each species’ data set to reduce erroneous hits due to sequencing errors (using the script ‘combine_HtSeq.pl’; Marx et al., 2020b).

We evaluated the performance of our TagSeq data in terms of recovery of reference loci, specificity of tag alignments, and variation in expression patterns among samples. To assess the ability of TagSeq to track loci in a reference transcriptome, we plotted the proportion of the reference sequences to which tags aligned as a function of TagSeq sequencing effort (total reads) and fitted a logarithmic curve to identify patterns of saturation with sequencing effort. To examine the specificity of the tags, we quantified the fraction of tags that mapped to multiple reference loci. We also compared the number of tags aligning to references when (i) the reference and TagSeq were derived from the same sample, (ii) the reference and tags were derived from different samples of the same species (individuals or populations), and (iii) the reference and tags were derived from different species.

Finally, we assessed expression differences among samples with MDS ordination of all TagSeq samples for a species. R/vegan version 2.4‐3 (Oksanen et al., 2016) was used to calculate the relative abundance matrix across loci and samples, and R/limma version 3.26.9 (Ritchie et al., 2015) was used to calculate root‐mean‐square deviation (Euclidean distance) among samples and construct the ordination. Distances were based on the loci with the largest standard deviation among all samples (gene.selection = “common”). The number of top loci used was determined by the median value of loci observed among samples: 60,000 for *B. aristidoides*, 54,000 for *E. lehmanniana*, 52,000 for *P. florida*, and 110,000 for *T. lanuginosa*.

## RESULTS

Raw data for RNA‐seq references and TagSeq gene expression were deposited at the National Center for Biotechnology Information (NCBI) Sequence Read Archive (BioProject #PRJNA599443). Reference sequencing included 64–83 million raw reads and 63–81 million clean reads, per species (Table 1). Assembly metrics indicated that the most complete assembly was obtained for *P. florida*, with 78% BUSCO recovery, N50 of 895 bp, and the largest fraction of contigs translating to known proteins (Table 1). The two grass species yielded the least comprehensive reference assemblies, with 45% and 49% BUSCO scores for *E. lehmanniana* and *B. aristidoides*, respectively, and N50 values below 600 bp for both species. Assembly metrics were generally intermediate for *T. lanuginosa*, although it had the largest number of assembled contigs and contigs translating to proteins. Notably, despite having the second largest sequencing effort, *E. lehmanniana* had the smallest maximum contig size, lowest BUSCO score, and fewest contigs matching known proteins, suggesting that contigs assembled more poorly for this species relative to the others.

**Table 1:** RNA‐seq reference assembly summary statistics for each species. Included are the numbers of raw reads, clean reads, assembled contigs, and contigs aligning to proteins (translating), as well as the N50 and maximum contig length (bp) and the percentage of BUSCO sequences matching contigs (complete and partial) in the Viridiplantae database.

Species	Raw reads	Clean reads	Assembled contigs	N50 bp (Max)	% BUSCO	Translating (%)
*Bouteloua aristidoides*	64,229,674	62,645,584	323,769	575 (5638)	49.4	25,952 (8.0)
*Eragrostis lehmanniana*	76,308,626	74,161,029	441,195	597 (3786)	45.4	18,830 (4.3)
*Parkinsonia florida*	83,770,528	81,442,270	348,947	895 (7054)	78.1	29,786 (8.5)
*Tidestromia lanuginosa*	69,670,835	69,670,835	1,035,859	647 (8009)	67.3	38,833 (3.7)

TagSeq libraries included a range of 2.6 million to 9.9 million raw reads per sample, except for two samples with low read counts: *E. lehmanniana* Sample 1 with 335,000 reads, and *T. lanuginosa* Sample 9 with 1.2 million reads (Appendix 2). Read cleaning resulted in a low proportion of reads removed due to quality issues (typically <10%). In contrast, PCR duplicates accounted for 42–61% of reads (for all samples other than Sample 1, for which PCR duplicates were 71% of reads).

Among the remaining unique tags, >80% of tags aligned to reference sequences for most samples, other than those of *E. lehmanniana*. For *E. lehmanniana*, 56–65% of tags aligned to the reference sequences (Appendix 2). The fraction of tags aligning to more than one reference was low across all samples (<1%), including those of the two tetraploid grass species. The fraction of the RNA‐seq reference sequences that were observed in TagSeq samples ranged from 10–24% (excluding Samples 1 and 9, which had low read counts), resulting in 33–45% of references observed across all samples together. Requiring that a tag be observed at least five times reduced the fraction of references observed by approximately half for each species.

**Figure 2:**
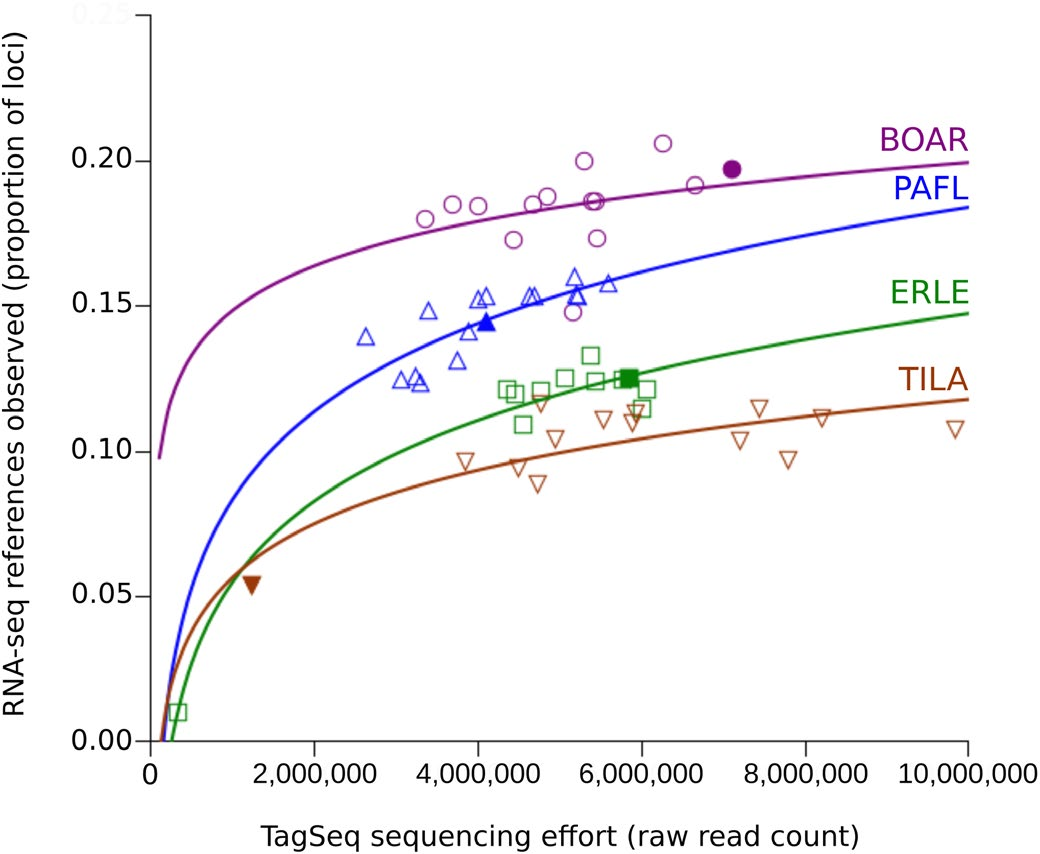
Proportion of RNA‐seq reference loci with aligned tags, as a function of sequencing effort (raw read number) of TagSeq libraries. Logarithmic best fits are shown for each species: *Bouteloua aristidoides* (BOAR, circles), *Eragrostis lehmanniana* (ERLE, squares), *Parkinsonia florida* (PAFL, upward triangles), and *Tidestromia lanuginosa* (TILA, downward triangles). Samples that were used for both a TagSeq library and the RNA‐seq reference are indicated by dark filled symbols. Two additional replicates of the reference *P. florida* individual collected on the same day are indicated by lightly shaded symbols. Reference loci were required to be observed in a minimum of five tags across a data set to be counted.

The number of reference sequences observed among tag sequences was related to the level of TagSeq sequencing effort (Fig. 2, Appendix 2). All species showed trends toward increases in the proportion of reference loci recovered with increasing sequencing effort, although all trends appeared to be saturating and additional sequencing was predicted to result in only modest increases in references observed. TagSeq samples that were the same as the RNA‐seq reference sample did not have disproportionately high matches to the reference sequence for their sequencing effort (Fig. 2); however, tag alignments to the reference sequence were highly species specific (Table 2). Between the two grass species (*B. aristidoides* and *E. lehmanniana*), 15–18% of tags aligned to the reference of the other species. For all other combinations of species, 7% or fewer tags aligned to a heterospecific reference.

**Table 2:** The proportion of tags from each sample (rows) aligning to each RNA‐seq reference (columns). Along the diagonal (shaded cells) are the proportion aligning to the conspecific reference for the sample, where the reference comes from a different individual (or different tissue collection of the same individual for *Parkinsonia florida*) collected at the same location and date. Off the diagonal are alignments of each sample to references from other species.

	RNA‐seq reference assembly			
Species (TagSeq sample no.)	*Bouteloua aristidoides*	*Eragrostis lehmanniana*	*Parkinsonia florida*	*Tidestromia lanuginosa*
*Bouteloua aristidoides* (54)	0.86	0.18	0.05	0.05
*Eragrostis lehmanniana* (49)	0.15	0.65	0.03	0.03
*Parkinsonia florida* (45)	0.05	0.05	0.89	0.07
*Tidestromia lanuginosa* (25)	0.03	0.03	0.03	0.85

**Figure 3:**
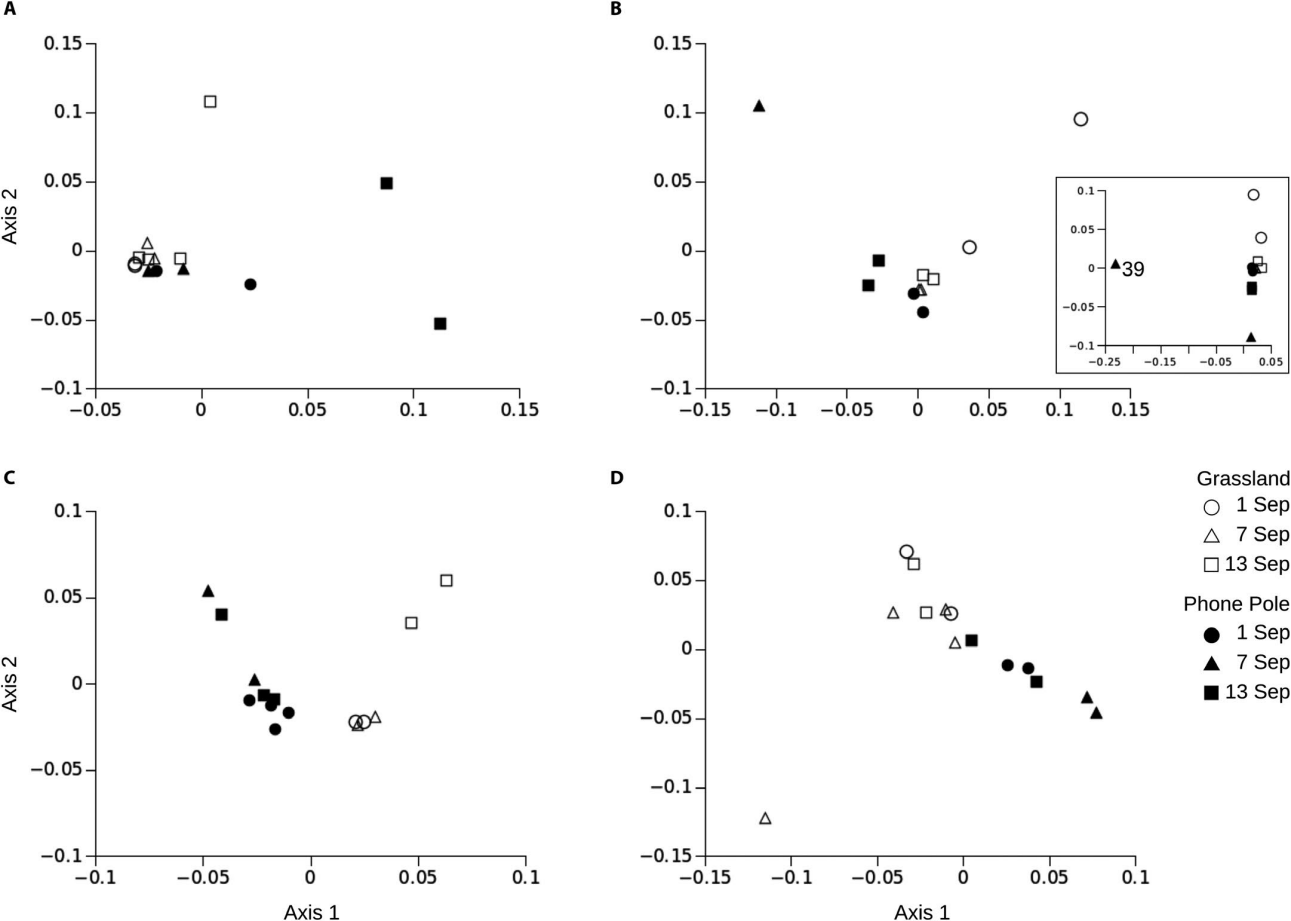
Multidimensional scaling (MDS) ordinations of TagSeq expression data for each species. Tissue samples for *Bouteloua aristidoides* (A), *Eragrostis lehmanniana* (B), *Parkinsonia florida* (C), and *Tidestromia lanuginosa* (D) were collected from two locations (Grassland, open symbols; Phone Pole, filled symbols) on three dates in 2017 (symbol shapes). Sample 39 for *E. lehmanniana* was highly divergent from others (B, inset) and was removed to better resolve variation among the remaining samples (B).

Ordinations for each species revealed clear variation in gene expression among samples (Fig. 3). All species showed clear separation between samples from different locations (closed vs. open symbols, Fig. 3). For *E. lehmanniana*, Sample 39 was strongly differentiated from all other samples along Axis 1 (Fig. 3B inset), and excluding this sample from the distance matrix allowed further resolution of variation among the remaining samples (Fig. 3B). Samples from different dates within a location had a weaker tendency to separate (different symbol shapes; Fig. 3), such that samples from the same location and date did not always cluster together.

## DISCUSSION

We evaluated the performance of TagSeq for surveys of gene expression in non‐model plant species, using repeated sampling of four species and alignment of tags to de novo assemblies of RNA‐seq reference transcriptomes. We found that a high fraction of tags aligned to each reference, and few tags mapped to multiple loci or to transcriptomes of other species. Samples from different locations showed clearly differentiated expression profiles for all four species, and these patterns were robust to sampling across three dates. Our results support the TagSeq approach as an effective means of generating specific and informative expression profiles in non‐model plants.

Quality filtering of tags resulted in very low losses of data (<11% of sequences for all but the most poorly sequenced sample in our data set), but PCR duplicates comprised 42–60% of samples. PCR duplicates are commonly abundant in Illumina library preparation methods (Aird et al., 2011), and Lohman et al. (2016) reported PCR duplicates of >70% for their test of the protocol used here. The large fraction of sequences involved in PCR duplicates emphasizes the importance of utilizing degenerate bases for identification and removal of duplicates when quantifying expression, as well as the importance of minimizing PCR cycles to maximize sequencing effort on sequences of interest.

For *E. lehmanniana*, >60% of filtered tags mapped to reference loci for most samples, and for nearly all remaining samples of the other species >80% of tags mapped to the reference. For the tags that did not map, at least three factors could explain their failure to align and the variation in alignment rates among species. First, reference loci must include the sequence at the 3' end of the transcript, immediately upstream of the poly(A) tail, where TagSeq reads will be located. RNA‐seq read distribution is random along the transcript, and therefore many loci will fail to include the required region by chance, and the fraction of loci lacking this region will vary among samples and with the sequencing effort used in creating the reference (Meyer et al., 2011; Conesa et al., 2016; Matz, 2018). Indeed, *E. lehmanniana* in particular showed evidence of having the least well‐assembled transcriptome among our references. Second, Lohman et al. (2016) found that TagSeq was more sensitive to low levels of expression than was RNA‐seq. This difference in sensitivity could result in novel low‐expression tags in the TagSeq data set, for which there is no representative locus in the RNA‐seq reference. Finally, allelic differences between samples could cause tags to fail to align to a reference sequence from another individual, although in our data set we did not see lower rates of alignment in samples that were different than that used for the RNA‐seq reference libraries.

For tags lacking a reference sequence, it would be possible in principle to cluster similar tags and to score their expression levels. We observed very low rates (<1%) of mapping to multiple reference loci, which suggests that clustering methods should be able to group tags into inferred loci without high rates of merging across different true loci. Without a reference sequence, however, no information would be available about the identity and function of those loci, which is typically the goal of expression studies (Conesa et al., 2016). Other references (e.g., annotated whole genomes of related species) could be explored for tag identification, but our analyses found that alignment rates to heterospecific loci were low (<20% within the same family, <10% between families).

From the perspective of the RNA‐seq reference library, a large fraction of reference loci (typically >80%) were not observed in individual TagSeq samples. Again, the samples used for both RNA‐seq and TagSeq did not recover a greater number of reference loci, suggesting that neither sequence differences between reference sequences and tags nor differences in genes expressed among samples explained the failure to observe a large number of reference loci in the tags. Additional TagSeq sequencing effort did not result in large gains in the observation of reference loci, although the combination of all samples roughly doubled the fraction of loci observed relative to any one sample, suggesting that tag sequencing effort within the range of our study will affect the number of loci observed. As described above, missing sequence information at the 3' end of reference loci will also have a large influence on alignment rates, and will set an upper limit on the fraction of loci that can be observed. In their initial publication of the next‐generation tag sequencing method, Meyer et al. (2011) also report that >80% of reference transcriptome sequences were poorly represented in their tag sequencing, and they suggest that this may be due to sequencing errors in the reference data set. Only 3.7–8.5% of our reference loci aligned to known proteins, and the number of loci translating to proteins was much more consistent with numbers of genes known from well‐studied genomes (Marx et al., 2020a), suggesting a large number of erroneous loci in our references. These issues regarding reference transcriptome quality could also explain differences in the maximum fraction of loci recovered among the different species.

Finally, we used ordinations to explore whether our resulting TagSeq expression data showed evidence of biologically relevant structure among samples, which would be amenable to further differential expression analyses. Our analyses revealed distinct separation of expression profiles between samples taken from different collection locations within each species. Spatial samples separated into non‐overlapping groups along the first (major) axis of ordinations for *P. florida* and *T. lanuginosa*, and through a combination of both axes for *B. aristidoides*. Spatial samples for *E. lehmanniana* converged for a few samples along axis 1. Temporal samples also appeared to group together within spatial locations for some combinations of dates, sites, and species, but additional sampling would be required to resolve temporal patterns robustly. Only one sample (Sample 39 for *E. lehmanniana*) across all species was an outlier in ordination space, such that it clustered far from the other samples and obscured variation in the remaining data set until it was removed.

In summary, we found that TagSeq expression profiles were biologically informative and showed little evidence of problems with tag specificity against non‐model transcriptome reference data sets. A large proportion of reference loci were not represented in the TagSeq data set, however, suggesting that completeness of reference assemblies (i.e., assembly of the 3' end) is likely to influence the identification of loci being expressed. Nevertheless, TagSeq quantified the expression of tens of thousands of loci for each species and revealed important patterns of differentiation among samples in our data set, suggesting that this is likely to be a fruitful approach for high‐throughput gene expression studies in non‐model plants.

## AUTHOR CONTRIBUTIONS

H.E.M., M.S.B, and K.M.D. conceived and designed the experiments. H.E.M. and S.S. collected and geolocated the samples. H.E.M. extracted the RNA and deposited the vouchers. K.M.D. and M.S.B. analyzed the data and drafted the manuscript. All authors contributed to the manuscript revision and approved the final version.

## Data Availability

Raw sequence data for RNA‐seq references and TagSeq gene expression have been deposited at the National Center for Biotechnology Information (NCBI) Sequence Read Archive under BioProject #PRJNA599443. RNA‐seq assemblies, translations, and all custom scripts are available at https://doi.org/10.5281/zenodo.3740232 (Marx et al., 2020b).

## Appendix 1 Sample collection site and date, NCBI Sequence Read Archive (SRA) accession, and information for vouchers deposited at the University of Arizona Herbarium (ARIZ).

**Table:**

Sample no.	Site	Latitude	Longitude	Date collected	SRA sample name	Herbarium accession no.^a^
*Bouteloua aristidoides*						
2*	Grassland	31.8695	−110.81533	13‐Sep‐17	Marx 2017‐074‐a‐09132017	
6	Phone Pole	31.88004	−110.89882	13‐Sep‐17	Marx 2017‐059‐a‐09132017	
20	Phone Pole	31.88004	−110.89882	1‐Sep‐17	Marx 2017‐003‐a‐09012017	435193
21	Phone Pole	31.88004	−110.89882	7‐Sep‐17	Marx 2017‐031‐a‐09072017	
23	Grassland	31.8695	−110.81533	1‐Sep‐17	Marx 2017‐018‐a‐09012017	435325
24	Grassland	31.8695	−110.81533	7‐Sep‐17	Marx 2017‐046‐a‐09072017	
27	Grassland	31.8695	−110.81533	1‐Sep‐17	Marx 2017‐018‐b‐09012017	435325
28	Grassland	31.8695	−110.81533	7‐Sep‐17	Marx 2017‐046‐b‐09072017	
32	Phone Pole	31.88004	−110.89882	1‐Sep‐17	Marx 2017‐003‐c‐09012017	435193
36	Phone Pole	31.88004	−110.89882	13‐Sep‐17	Marx 2017‐059‐c‐09132017	
41	Phone Pole	31.88004	−110.89882	7‐Sep‐17	Marx 2017‐031‐b‐09072017	
51	Grassland	31.8695	−110.81533	13‐Sep‐17	Marx 2017‐074‐b‐09132017	
54	Grassland	31.8695	−110.81533	13‐Sep‐17	Marx 2017‐074‐c‐09132017	
56	Grassland	31.8695	−110.81533	13‐Sep‐17	Marx 2017‐074‐d‐09132017	
*Eragrostis lehmanniana*						
1	Grassland	31.868985	−110.81573	13‐Sep‐17	Marx 2017‐084‐a‐09132017	
3	Phone Pole	31.88004	−110.89882	7‐Sep‐17	Marx 2017‐032‐a‐09072017	
10*	Grassland	31.868985	−110.81573	1‐Sep‐17	Marx 2017‐028‐a‐09012017	435319
15	Grassland	31.868985	−110.81573	7‐Sep‐17	Marx 2017‐056‐a‐09072017	
16	Phone Pole	31.88004	−110.89882	13‐Sep‐17	Marx 2017‐060‐a‐09132017	
17	Phone Pole	31.88004	−110.89882	1‐Sep‐17	Marx 2017‐004‐a‐09012017	435194
26	Grassland	31.868985	−110.81573	7‐Sep‐17	Marx 2017‐056‐b‐09072017	
30	Grassland	31.868985	−110.81573	13‐Sep‐17	Marx 2017‐084‐b‐09132017	
39	Phone Pole	31.88004	−110.89882	7‐Sep‐17	Marx 2017‐032‐b‐09072017	
43	Phone Pole	31.88004	−110.89882	13‐Sep‐17	Marx 2017‐060‐b‐09132017	
44	Phone Pole	31.88004	−110.89882	1‐Sep‐17	Marx 2017‐004‐b‐09012017	435194
49	Grassland	31.868985	−110.81573	1‐Sep‐17	Marx 2017‐028‐b‐09012017	435319
*Parkinsonia florida*						
11	Phone Pole	31.880457	−110.89868	1‐Sep‐17	Marx 2017‐014‐a‐09012017	435322
12*	Phone Pole	31.880457	−110.89868	13‐Sep‐17	Marx 2017‐070‐a‐09132017	
13	Grassland	31.869053	−110.81528	7‐Sep‐17	Marx 2017‐051‐b‐09072017	
14	Grassland	31.869053	−110.81528	1‐Sep‐17	Marx 2017‐023‐b‐09012017	435326
19	Phone Pole	31.880457	−110.89868	1‐Sep‐17	Marx 2017‐014‐b‐09012017	435322
22	Phone Pole	31.880457	−110.89868	7‐Sep‐17	Marx 2017‐042‐a‐09072017	
31	Grassland	31.869053	−110.81528	7‐Sep‐17	Marx 2017‐051‐c‐09072017	
34	Grassland	31.869053	−110.81528	13‐Sep‐17	Marx 2017‐079‐b‐09132017	
37	Grassland	31.869053	−110.81528	13‐Sep‐17	Marx 2017‐079‐c‐09132017	
38	Phone Pole	31.880457	−110.89868	13‐Sep‐17	Marx 2017‐070‐b‐09132017	
40	Phone Pole	31.880457	−110.89868	7‐Sep‐17	Marx 2017‐042‐b‐09072017	
45	Phone Pole	31.880457	−110.89868	13‐Sep‐17	Marx 2017‐070‐c‐09132017	
46	Phone Pole	31.880457	−110.89868	1‐Sep‐17	Marx 2017‐014‐c‐09012017	435322
50	Phone Pole	31.880457	−110.89868	1‐Sep‐17	Marx 2017‐014‐d‐09012017	435322
52	Grassland	31.869053	−110.81528	7‐Sep‐17	Marx 2017‐051‐d‐09072017	
55	Grassland	31.869053	−110.81528	1‐Sep‐17	Marx 2017‐023‐d‐09012017	435326
*Tidestromia lanuginosa*						
4	Phone Pole	31.879973	−110.89881	1‐Sep‐17	Marx 2017‐008‐a‐09012017	435324
5	Phone Pole	31.879973	−110.89881	13‐Sep‐17	Marx 2017‐064‐a‐09132017	
7	Grassland	31.869432	−110.81542	13‐Sep‐17	Marx 2017‐072‐a‐09132017	
8	Phone Pole	31.879973	−110.89881	7‐Sep‐17	Marx 2017‐036‐a‐09072017	
9*	Grassland	31.869432	−110.81542	7‐Sep‐17	Marx 2017‐043‐a‐09072017	
18	Grassland	31.869432	−110.81542	1‐Sep‐17	Marx 2017‐016‐a‐09012017	435323
25	Grassland	31.869432	−110.81542	7‐Sep‐17	Marx 2017‐043‐b‐09072017	
29	Grassland	31.869432	−110.81542	13‐Sep‐17	Marx 2017‐072‐b‐09132017	
33	Phone Pole	31.879973	−110.89881	1‐Sep‐17	Marx 2017‐008‐b‐09012017	435324
35	Phone Pole	31.879973	−110.89881	7‐Sep‐17	Marx 2017‐036‐b‐09072017	
42	Grassland	31.869432	−110.81542	1‐Sep‐17	Marx 2017‐016‐b‐09012017	435323
47	Phone Pole	31.879973	−110.89881	13‐Sep‐17	Marx 2017‐064‐b‐09132017	
48	Grassland	31.869432	−110.81542	7‐Sep‐17	Marx 2017‐043‐c‐09072017	
53	Grassland	31.869432	−110.81542	7‐Sep‐17	Marx 2017‐043‐d‐09072017	

^a^ Accession numbers are provided for herbarium vouchers associated with an RNA sample from the same plant (additional RNA samples from the same species and location are not vouchered).

* Samples used as both RNA‐seq reference transcriptome libraries and TagSeq expression libraries.

## Appendix 2 TagSeq summary statistics for each sample.

**Table:** Note

	Count:	Count:	Proportion of reads removed due to:			Count:	Proportion of tags:			Proportion of ref. contigs:	
Sample no.	Raw reads	Clean tags	PCR duplicates	Short	Cont./Qual.	Tags aligned	Aligned	Not aligned	Mult. hits	Total hits	Min. 5 hits
*Bouteloua aristidoides*											
2*	7,108,820	2,864,252	0.54	0.0001	0.05	2,420,723	0.85	0.15	0.004	0.22	0.20
6	6,659,522	2,617,957	0.56	0.0001	0.05	2,079,881	0.79	0.21	0.005	0.22	0.19
20	5,317,488	2,455,245	0.47	0.0001	0.06	2,045,210	0.83	0.17	0.004	0.23	0.20
21	5,163,416	2,201,758	0.52	0.0000	0.05	1,096,620	0.50	0.50	0.003	0.16	0.15
23	5,464,515	2,214,799	0.53	0.0001	0.06	1,879,440	0.85	0.15	0.005	0.19	0.17
24	4,684,318	2,176,801	0.48	0.0001	0.06	1,754,713	0.81	0.19	0.004	0.20	0.18
27	4,451,466	1,939,050	0.50	0.0001	0.06	1,643,622	0.85	0.15	0.004	0.19	0.17
28	4,849,566	2,143,817	0.50	0.0001	0.06	1,810,488	0.84	0.16	0.005	0.21	0.19
32	5,438,574	2,415,989	0.50	0.0001	0.05	2,024,038	0.84	0.16	0.004	0.20	0.19
36	6,268,774	2,748,050	0.50	0.0001	0.06	2,284,930	0.83	0.17	0.005	0.24	0.21
41	5,405,364	2,246,322	0.53	0.0001	0.05	1,859,298	0.83	0.17	0.005	0.21	0.19
51	3,695,704	1,751,372	0.45	0.0001	0.07	1,471,954	0.84	0.16	0.005	0.21	0.18
54	3,355,992	1,696,597	0.42	0.0002	0.07	1,455,374	0.86	0.14	0.004	0.20	0.18
56	4,009,876	1,900,705	0.46	0.0001	0.07	1,629,273	0.86	0.14	0.004	0.20	0.18
TOTAL		31,372,714								0.45	0.27
*Eragrostis lehmanniana*											
1	335,464	17,904	0.71	0.0073	0.22	10,362	0.58	0.42	0.006	0.01	0.01
3	6,071,239	2,538,484	0.53	0.0001	0.05	1,592,331	0.63	0.37	0.005	0.14	0.12
10*	5,866,891	2,366,892	0.55	0.0000	0.04	1,522,526	0.64	0.36	0.006	0.15	0.12
15	6,013,007	2,267,378	0.58	0.0000	0.04	1,457,729	0.64	0.36	0.005	0.13	0.11
16	5,779,005	2,374,148	0.54	0.0001	0.05	1,542,273	0.65	0.35	0.005	0.15	0.12
17	5,440,165	2,303,471	0.52	0.0000	0.05	1,427,988	0.62	0.38	0.005	0.14	0.12
26	4,772,169	2,096,114	0.50	0.0001	0.06	1,374,445	0.66	0.34	0.005	0.14	0.12
30	4,458,162	1,968,870	0.50	0.0001	0.06	1,293,357	0.66	0.34	0.006	0.14	0.12
39	5,386,577	2,705,308	0.44	0.0001	0.05	1,520,821	0.56	0.44	0.004	0.17	0.13
43	5,067,830	2,199,399	0.51	0.0001	0.06	1,401,928	0.64	0.36	0.005	0.15	0.13
44	4,373,470	1,993,560	0.49	0.0000	0.06	1,209,574	0.61	0.39	0.005	0.14	0.12
49	4,559,967	1,803,395	0.56	0.0001	0.04	1,169,445	0.65	0.35	0.006	0.12	0.11
TOTAL		24,634,923								0.33	0.17
*Parkinsonia florida*											
11	3,887,959	1,432,767	0.53	0.0003	0.10	1,253,182	0.87	0.13	0.005	0.15	0.14
12*	4,110,903	1,603,854	0.55	0.0001	0.06	1,444,493	0.90	0.10	0.005	0.16	0.14
13	3,305,618	1,146,180	0.58	0.0002	0.08	992,508	0.87	0.13	0.005	0.13	0.12
14	3,066,122	1,155,170	0.58	0.0001	0.04	982,613	0.85	0.15	0.005	0.13	0.12
19	5,207,037	1,779,503	0.60	0.0001	0.06	1,607,565	0.90	0.10	0.005	0.17	0.15
22	4,643,217	1,704,905	0.56	0.0001	0.07	1,524,181	0.89	0.11	0.005	0.17	0.15
31	3,250,480	1,140,899	0.59	0.0001	0.06	1,010,075	0.89	0.11	0.005	0.13	0.13
34	4,694,661	1,931,488	0.52	0.0003	0.07	1,697,593	0.88	0.12	0.005	0.17	0.15
37	5,231,677	2,026,252	0.54	0.0002	0.07	1,772,085	0.87	0.13	0.005	0.17	0.15
38	5,194,831	2,064,010	0.53	0.0002	0.07	1,864,999	0.90	0.10	0.005	0.18	0.16
40	5,607,535	2,098,556	0.55	0.0002	0.07	1,856,128	0.88	0.12	0.004	0.17	0.16
45	4,106,462	1,598,327	0.54	0.0001	0.07	1,427,567	0.89	0.11	0.005	0.17	0.15
46	3,395,667	1,362,410	0.52	0.0001	0.07	1,224,492	0.90	0.10	0.005	0.16	0.15
50	4,011,175	1,550,240	0.55	0.0001	0.07	1,400,355	0.90	0.10	0.005	0.17	0.15
52	2,638,261	1,165,622	0.46	0.0005	0.09	1,010,315	0.87	0.13	0.005	0.16	0.14
55	3,761,388	1,348,379	0.56	0.0001	0.08	1,168,972	0.87	0.13	0.005	0.14	0.13
TOTAL		25,108,562								0.36	0.21
*Tidestromia lanuginosa*											
4	8,206,366	3,314,540	0.55	0.0001	0.04	2,698,441	0.81	0.19	0.005	0.13	0.11
5	7,206,798	2,879,104	0.56	0.0000	0.04	2,359,448	0.82	0.18	0.005	0.12	0.10
7	7,803,507	2,732,931	0.61	0.0001	0.04	2,266,700	0.83	0.17	0.004	0.11	0.10
8	9,851,038	3,308,006	0.63	0.0000	0.03	2,723,453	0.82	0.18	0.005	0.13	0.11
9*	1,241,373	555,457	0.45	0.0021	0.11	436,457	0.79	0.21	0.004	0.06	0.05
18	4,744,115	1,982,995	0.53	0.0000	0.06	1,538,724	0.78	0.22	0.004	0.10	0.09
25	5,542,647	2,477,967	0.49	0.0001	0.06	2,100,990	0.85	0.15	0.005	0.14	0.11
29	5,944,645	2,594,919	0.52	0.0000	0.05	2,155,507	0.83	0.17	0.005	0.14	0.11
33	5,891,911	2,708,682	0.49	0.0001	0.05	2,281,047	0.84	0.16	0.005	0.13	0.11
35	7,448,586	3,111,221	0.53	0.0001	0.05	2,559,379	0.82	0.18	0.004	0.14	0.11
42	4,957,036	2,249,422	0.49	0.0001	0.06	1,812,268	0.81	0.19	0.004	0.13	0.10
47	4,508,978	1,871,639	0.53	0.0000	0.06	1,563,872	0.84	0.16	0.005	0.11	0.09
48	3,857,432	1,822,810	0.46	0.0001	0.07	1,544,443	0.85	0.15	0.004	0.11	0.10
53	4,770,648	2,282,278	0.47	0.0001	0.06	1,884,754	0.83	0.17	0.004	0.14	0.12
TOTAL		33,891,971								0.37	0.19

Cont./Qual. = tags removed due to contamination or low‐quality scores; Mult. hits = hits to multiple reference loci.

* Samples used as both RNA‐seq reference transcriptome libraries and TagSeq expression libraries.
